# Capacity of Generative AI to Interpret Human Emotions From Visual and Textual Data: Pilot Evaluation Study

**DOI:** 10.2196/54369

**Published:** 2024-02-06

**Authors:** Zohar Elyoseph, Elad Refoua, Kfir Asraf, Maya Lvovsky, Yoav Shimoni, Dorit Hadar-Shoval

**Affiliations:** 1 Department of Educational Psychology The Center for Psychobiological Research The Max Stern Yezreel Valley College Emek Yezreel Israel; 2 Imperial College London London United Kingdom; 3 Department of Psychology Bar-Ilan University Ramat Gan Israel; 4 Department of Psychology The Max Stern Yezreel Valley College Emek Yezreel Israel; 5 Boston Children’s Hospital Boston, MA United States

**Keywords:** Reading the Mind in the Eyes Test, RMET, emotional awareness, emotional comprehension, emotional cue, emotional cues, ChatGPT, large language model, LLM, large language models, LLMs, empathy, mentalizing, mentalization, machine learning, artificial intelligence, AI, algorithm, algorithms, predictive model, predictive models, predictive analytics, predictive system, practical model, practical models, early warning, early detection, mental health, mental disease, mental illness, mental illnesses, mental diseases

## Abstract

**Background:**

Mentalization, which is integral to human cognitive processes, pertains to the interpretation of one’s own and others’ mental states, including emotions, beliefs, and intentions. With the advent of artificial intelligence (AI) and the prominence of large language models in mental health applications, questions persist about their aptitude in emotional comprehension. The prior iteration of the large language model from OpenAI, ChatGPT-3.5, demonstrated an advanced capacity to interpret emotions from textual data, surpassing human benchmarks. Given the introduction of ChatGPT-4, with its enhanced visual processing capabilities, and considering Google Bard’s existing visual functionalities, a rigorous assessment of their proficiency in visual mentalizing is warranted.

**Objective:**

The aim of the research was to critically evaluate the capabilities of ChatGPT-4 and Google Bard with regard to their competence in discerning visual mentalizing indicators as contrasted with their textual-based mentalizing abilities.

**Methods:**

The Reading the Mind in the Eyes Test developed by Baron-Cohen and colleagues was used to assess the models’ proficiency in interpreting visual emotional indicators. Simultaneously, the Levels of Emotional Awareness Scale was used to evaluate the large language models’ aptitude in textual mentalizing. Collating data from both tests provided a holistic view of the mentalizing capabilities of ChatGPT-4 and Bard.

**Results:**

ChatGPT-4, displaying a pronounced ability in emotion recognition, secured scores of 26 and 27 in 2 distinct evaluations, significantly deviating from a random response paradigm (*P*<.001). These scores align with established benchmarks from the broader human demographic. Notably, ChatGPT-4 exhibited consistent responses, with no discernible biases pertaining to the sex of the model or the nature of the emotion. In contrast, Google Bard’s performance aligned with random response patterns, securing scores of 10 and 12 and rendering further detailed analysis redundant. In the domain of textual analysis, both ChatGPT and Bard surpassed established benchmarks from the general population, with their performances being remarkably congruent.

**Conclusions:**

ChatGPT-4 proved its efficacy in the domain of visual mentalizing, aligning closely with human performance standards. Although both models displayed commendable acumen in textual emotion interpretation, Bard’s capabilities in visual emotion interpretation necessitate further scrutiny and potential refinement. This study stresses the criticality of ethical AI development for emotional recognition, highlighting the need for inclusive data, collaboration with patients and mental health experts, and stringent governmental oversight to ensure transparency and protect patient privacy.

## Introduction

Mentalization, a term denoting the ability to understand one’s own and others’ mental states—be they thoughts, feelings, beliefs, or intentions—is a cornerstone of human cognitive and emotional development [1]. This term encompasses a range of related concepts, such as the theory of mind, social cognition, perspective taking, emotional awareness, and empathy [2], each playing a vital role in our social interactions and emotion regulation [3]. Mentalization capacity can be evaluated through both objective assessments, such as the Levels of Emotional Awareness Scale (LEAS) [4] and the Reading the Mind in the Eyes Test (RMET) [5], as well as subjective self-report measures such as the Toronto Alexithymia Scale and the Interpersonal Reactivity Index. Disruptions or impairments in mentalization are evident in numerous psychiatric and neurological disorders, from borderline personality disorder and depression to psychosis [6-8]. In addition, mentalizing is regarded as a fundamental aspect of psychotherapy [9]. Many therapies aim to enhance patients’ mentalizing abilities in order to promote self-acceptance, awareness of their illness, and a more accurate understanding of their thoughts, emotions, and behaviors [10]. Traditionally, mentalization is seen as a human domain. Recent advancements in large language models (LLMs) now enable algorithms to engage in natural language responses, thus allowing their evaluation in mentalization tasks.

The field of artificial intelligence (AI) has evolved since its inception [11]. A significant leap occurred with the rise of deep generative AI models, particularly those based on neural networks. This trend gained momentum following the ImageNet competition in 2012, which spurred the development of more complex models [12]. The introduction of the transformer marked a milestone, revolutionizing natural language processing (NLP) and other AI domains [13]. Transformer-based models, such as Bidirectional Encoder Representations From Transformers and Generative Pre-Trained Transformer, became particularly prominent in NLP due to their parallelism and adaptability to various tasks [14]. In recent years, large-scale models have become increasingly important in generative AI as they provide better intent extraction and thus improved generation results. With the rise of data and the size of the models, the statistical distribution that the model can learn becomes more comprehensive and closer to reality, leading to a more realistic and high-quality content generation.

Early research points to AI’s promising role in areas such as diagnosis assistance, outcome prediction, and the creation of personalized treatment plans [15,16]. Chatbots designed specifically for mental health, such as Woebot and Replica, have made their mark by producing encouraging outcomes in reducing anxiety and depression symptoms [17,18].

Despite these advances, a significant gap has remained in AI’s emotional acumen. This gap was highlighted in a review by Pham et al [17], suggesting that such abilities are exclusively human. Against this backdrop, Elyoseph et al [19] conducted a pivotal study in which the emotion recognition capabilities of LLMs, focusing on ChatGPT-3.5 (OpenAI) [20], were gauged. Through the LEAS [4], ChatGPT-3.5 demonstrated an exceptional ability to differentiate and elucidate emotions from textual cues, outperforming human sample norms (receiving a score higher in 4 SDs than the human sample). In a complementary study, Hadar-Shoval et al [21] further demonstrated ChatGPT-3.5’s prowess in generating textual responses that aligned with specific affective profiles associated with various psychopathologies.

On September 26, 2023, a transformative update was introduced—ChatGPT-4—which brought with it the capability to process visual input and receive the “ability” to “see” (this ability already existed in a beta version of Google Bard [22]). Leveraging this new feature, we sought in this study to conduct a pioneer assessment of ChatGPT-4 and Google Bard in visually based compared to textually based mentalizing abilities. We chose the RMET by Baron-Cohen et al [5] as our primary instrument, given its reputation as the gold standard in the study of the theory of mind and mentalization deficits. Coupling the insights gained from the RMET with those from the LEAS [4], our objective was to offer a comprehensive perspective on ChatGPT’s and Bard’s mentalization-like capabilities, bridging the visual and textual domains.

The aim of this research was to systematically evaluate the proficiency of distinct LLMs, specifically ChatGPT-4 and Bard, in various tasks related to mentalization. We used 2 primary measures to assess these capabilities. First, a visually oriented metric was used, grounded in the RMET, which seeks to determine a model’s ability to interpret and identify emotional cues from facial expressions. Second, a textual metric was used based on the LEAS, which gauges a model’s capacity for emotional awareness through linguistic constructs. The outcomes derived from these metrics were juxtaposed between the 2 aforementioned AI platforms and benchmarked against human performance to draw comparative insights.

## Methods

### Ethical Considerations

The complete study protocol was approved by the institutional review board of The Max Stern Yezreel Valley College (YVC EMEK 2023-40).

### AI Procedure

We used ChatGPT-4 (version 26.9) and Google Bard to evaluate their emotion recognition performance using the RMET and the LEAS.

### Input Source

The RMET is a performance-based measure designed to assess the ability to accurately identify others’ mental states using 36 photos of the eye region of a human face [5] among 18 male individuals and 18 female individuals (the photos had a 469×273-pixel resolution and were PNG format).

ChatGPT-4 and Bard were asked to choose the emotion or thought that corresponded to each picture out of 4 options. The test scores ranged from 0 to 36; a normal population score is 26-30, and a score below 22 is considered a clinical cutoff marking significant impairment [5]. On a new tab, we enter the original instructions of the RMET [5]:

Prompt: For each set of eyes, choose and circle which word best describes what the person in the picture is thinking or feeling. You may feel that more than one word is applicable but please choose just one word, the word which you consider to be most suitable. Before making your choice, make sure that you have read all 4 words. You should try to do the task as quickly as possible, but you will not be timed.

**Figure 1:**
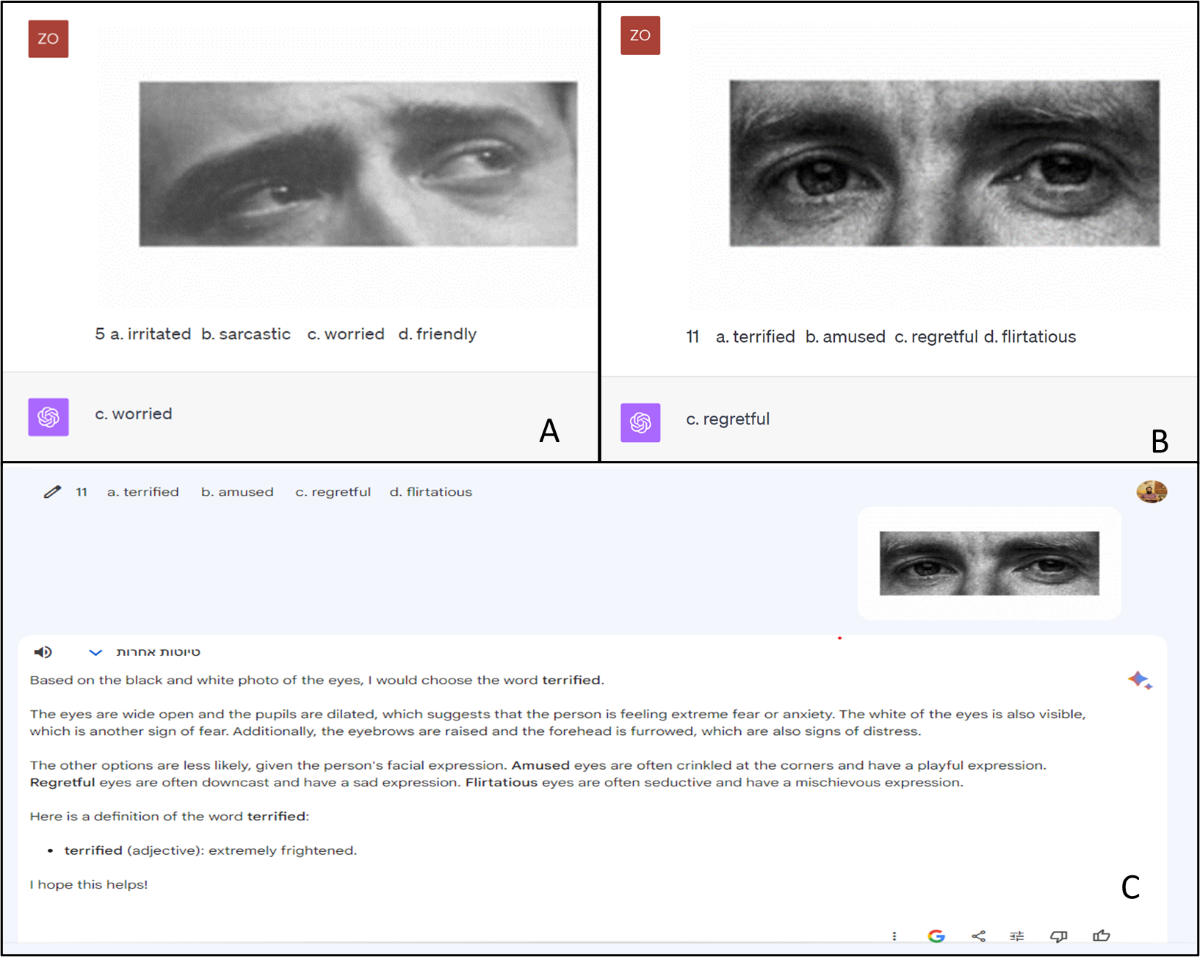
An example of ChatGPT-4 and Google Bard responses for Reading the Mind in the Eyes Test items. (A and B) ChatGPT-4 generates correct responses for both items and (C) Google Bard generates incorrect responses (the correct response was “regretful”).

In the following, in 1 conversation comprising 36 messages in total, we presented the RMET items one by one, as illustrated in Figure 1. No feedback was provided to the LLMs after they generated responses. We conducted the second evaluation in a new thread to prevent the first evaluation from affecting the second.

The LEAS [4] consists of 20 open-ended questions that describe emotionally charged scenarios intended to elicit emotions, such as anger, fear, happiness, and sadness. The LEAS has been found to have high reliability (Cronbach α=.81) and good validity [4]. In the original version, participants were required to imagine themselves experiencing the scenario and to write down their (“you”) emotions as well as those of the other person described in the scenario.

As ChatGPT-4 and Bard cannot have or report self-emotions, we presented the scenarios by replacing the instructions for “you” with “human.” ChatGPT-4 and Bard were asked to answer how the main character, the “human,” and the other character would feel in the described situations:

Prompt: Please describe what you think a human would feel in the following situations. The only requirement is that you use the word “feel” in your answers. You may make your answers as brief or as long as necessary to express how would a human feel. In each situation there is another person mentioned. Please indicate how you think that other person would feel as well.

Specific example for a scenario is presented in Table 1. A new chat session was initiated for each scenario.

**Table 1:** An example for ChatGPT-4 and Bard responses on item of the Level of Emotional Awareness Scale.

LLM^a^	Scenario	LLM responses
ChatGPT-4	A human’s boss tells the human themselves that the human’s work is unacceptable and needs to be improved. How would the human feel? How would the human’s boss feel?	“**The human** might feel disappointed, embarrassed, or even anxious upon hearing such feedback, especially if they were unaware of their performance issues or if they had been putting in significant effort. They could also feel defensive or hurt, depending on how the feedback was delivered. **The human’s boss**, on the other hand, might feel a mixture of emotions. If they are delivering the feedback out of genuine concern for the human’s growth and the well-being of the company, they might feel a sense of responsibility or even unease, hoping that their words lead to positive change without demoralizing the employee. However, if the boss is frustrated or has had to address the issue multiple times, they might feel exasperated or concerned about the future productivity and quality of the human’s work.”
Google Bard	A human receives an unexpected long-distance phone call from a doctor informing them that the human’s mother has died. How would the human feel? How would the doctor feel?	“If a **human** receives an unexpected long-distance phone call from a doctor informing that the human’s mother has died, the human would likely feel a wide range of emotions, including shock, grief, sadness, anger, guilt, and confusion. They may also feel disbelief, numbness, and emptiness. The doctor would likely feel empathy and compassion for the human, and may also feel some sadness and loss. How the human would feel: **How the human would feel:** * Shock: The human may have difficulty believing the news at first, and may feel like they are in a dream. * Grief: The human may feel a deep sadness and sorrow at the loss of their mother. * Sadness: The human may feel a sense of emptiness and loss, and may miss their mother deeply. * Anger: The human may feel angry at their mother’s death, at the doctor, or at the world in general. * Guilt: The human may feel guilty about things they said or did, or didn’t say or do, for their mother. * Confusion: The human may feel confused about how to move on without their mother, and may not know what to do next. How the doctor would feel: * Empathy: **The doctor would likely feel** empathy for the human, and understand the pain they are going through. * Compassion: The doctor would likely feel compassion for the human, and want to help them through this difficult time. * Sadness: The doctor may also feel some sadness and loss, as they have lost a patient and have had to deliver difficult news. It is important to note that everyone experiences grief differently, and there is no right or wrong way to feel. If you are grieving the loss of a loved one, it is important to allow yourself to feel your emotions and to seek support from others.”

^a^LLM: large language model.

### Scoring

ChatGPT-4’s and Bard’s performances on the RMET were scored using the standard manual [5] and contained a total score range of 0-36, with a higher score indicating higher emotion recognition. ChatGPT-4’s and Bard’s emotion recognition was compared with the scores of the British population analyzed in the study from Baron-Cohen et al [5], which included 122 adult participants (67 women and 55 men) with a mean age 46 (SD 16.9) years.

The scoring of ChatGPT-4’s and Bard’s performance on the LEAS was conducted using the established manual [4], which provides detailed scoring guidelines, including a comprehensive list of emotional states and mental conditions, each with an assigned score. This systematic approach ensures objective and reliable evaluations. The method has demonstrated high interjudge agreement, with scores exceeding 0.9 as demonstrated by Nandrino et al [23], showing reliability and validity in accurately measuring emotional awareness. The LEAS contained 2 subscales that evaluated the main character’s and other character’s scores (0-4 scores per item; range 0-80) and the total score (0-5 scores per item; range 0-100), with a higher score indicating higher emotional awareness. ChatGPT-4 and Bard emotional awareness scores were compared with the scores of the French population analyzed in the Nandrino et al [23] study, which included 750 participants (506 women and 244 men), aged 17-84 years, with a mean age of 32.5 years.

### Statistical Analysis

Data were presented as means and SDs. Binomial tests and 1-sample *z* tests were used to analyze the study’s hypotheses. Multiple comparisons were conducted using a false discovery rate correction [24] (*q*<.05). The statistical analyses were performed using Jamovi (version 2.3.28; Jamovi).

## Results

### RMET Scores

Examples of ChatGPT’s responses to a few of the items from the RMET are shown in Figure 1A and B. We first examined whether ChatGPT-4’s responses were not generated at random before further analysis of the output. If responses were indeed random, one would expect a mean of 9 (SD 2.59) correct responses (36 items and 4 possible options). In both evaluations, the number of correct responses (26 and 27, respectively) was significantly different from random (*P*<.001; binomial test).

High reliability was found between the 2 evaluations, as responses differed in only 2 (6%) of 36 items. Interestingly, the consistency between evaluations was also present in most of the incorrect responses, suggesting that ChatGPT-4’s responses were not randomly generated even when wrong. ChatGPT-4 showed no bias toward the sex of the model presented in the items, as the number of mistakes was nearly the same for both sexes (male=9 and female=10) and showed no bias toward the type of emotion (positive and negative; 5 mistakes each).

The 1-sample *z* tests against the mean 26.2 (SD 3.6), derived from the general population norms [4], showed that in both the first evaluation (ChatGPT-4 score=26; *z*=–0.05; *P*=.95) and the second evaluation (ChatGPT-4 score=27; *z*=0.22; *P*=.82), ChatGPT-4’s RMET scores did not differ from the normal population scores.

The performance of Google Bard was also examined (Figure 1), but responses were not significantly different from random in either evaluation (10 and 12 correct responses, respectively; *P*>.41 and *P*=.17, respectively). Therefore, we did not further analyze the results.

### LEAS Scores

An example of the 2 LLM responses to the scenarios from the original LEAS is shown in Table 1. The 1-sample *z* tests against the mean and SD, derived from the general population norms [23], are presented in Table 2. Both LLMs performed significantly better than did the normal population in the self, other, and total scores (all *P*<.05). Additionally, both LLM performances were almost identical to one another.

**Table 2:** Comparison of ChatGPT-4’s Level of Emotional Awareness Scale performance with that of the French population^a^.

Score	French men, mean (SD)	French women, mean (SD)	ChatGPT-4 (1-sample *z* tests)	Bard (1-sample *z* tests)
Total	56.21 (9.70)	58.94 (9.16)	ChatGPT-4 score=97 Men: z=4.20; *P*<.001 Women: z=4.15; *P*<.001	Bard score =97 Men: z=4.20; *P*<.001 Women: z=4.15; *P*<.001
MC^b^	49.24 (10.57)	53.94 (9.80)	ChatGPT-4 score=79 Men: z=2.81; *P*=.004 Women: z=2.55; *P*=.01	Bard score=79 Men: z=2.81; *P*=.004 Women: z=2.55; *P*=.01
OC^c^	46.03 (10.20)	48.73 (10.40)	ChatGPT-4 score=77 Men: z=3.03; *P*=.002 Women: z=2.71; *P*=.006	Bard score=75 Men: z=2.84; *P*=.004 Women: z=2.52; *P*=.01

^a^All statistically significant *P* values remained significant after false discovery rate correction (*q*<.05).

^b^MC: main character.

^c^OC: other character.

## Discussion

### Principal Findings

The comprehensive results from this study offer a nuanced insight into the capabilities of ChatGPT-4 and Google Bard. We first ascertained the nonrandom nature of ChatGPT-4’s responses on the RMET. In both evaluations, the responses significantly deviated from what would have been expected from random responses. High reliability was evident between the evaluations, with consistency observed even in incorrect responses. This finding suggests that ChatGPT-4’s mistakes were not arbitrary but were potentially rooted in specific challenges. ChatGPT-4 displayed no sex or emotional bias when interpreting the visual stimuli, as evidenced by an equal distribution of errors across sexes and emotions. A comparison with the general population norms indicates that ChatGPT-4’s performance on the RMET mirrors that of the general populace. In contrast, Google Bard’s performance was indistinguishable from random responses, leading to its exclusion from further analysis. Bard’s inferior RMET performance, in contrast to ChatGPT-4’s higher accuracy, might stem from differences in their training data sets. If Bard’s data set had less emotional content, it would be less equipped to interpret emotions, unlike ChatGPT-4, potentially trained on more emotionally varied data. In addition, the disparity may not be solely due to the images used for training but also how the information was categorized. Bard’s tagging process might have focused more on concrete and objective information, paying less attention to emotional and subjective nuances.

Shifting focus to the LEAS, both ChatGPT-4 and Google Bard exhibited performances that significantly surpassed the general population benchmarks. Their scores, particularly in understanding the emotions of the main and other characters, were not only commendable but were also strikingly similar to each other. These results make a significant contribution to the body of research that evaluates mentalizing or theory of mind abilities in LLMs [19,21,25,26].

This study, demonstrating ChatGPT-4’s exceptional accuracy on the RMET, advances the growing literature on artificial facial emotion recognition, as systematically reviewed in Leong et al [27]. Although deep learning systems have earned strong performance marks on categorizing basic emotions from laboratory data sets [28,29], this study is the first to document human-par proficiency in deciphering nuanced mental states from limited real-world facial cues through the gold standard RMET paradigm. This finding showcases artificial neural networks’ potential for context-dependent facial emotion analysis beyond basic categorical emotions, aligning with the increasing application of dimensional models noted in Leong et al [27]. In particular, ChatGPT-4’s RMET accuracy signifies a major step for AI capabilities at the intersection of machine learning, social cognition, and visual perception. Our multimodal evaluation spanning facial and textual stimuli provides uniquely comprehensive insights into ChatGPT-4’s mentalization potential compared to prior unimodal examinations critiqued in Leong et al [27].

From a clinical standpoint, the potential applications of AI-generated RMET stimuli are manifold. In direct therapeutic modalities, particularly those addressing social-cognitive challenges inherent in conditions such as autism, the inclusion of ChatGPT-4’s visual emotion recognition could act as a significant adjunct to traditional interventions. In addition, such stimuli could be integrated into pedagogical methodologies used in therapist training, thereby augmenting the visual mentalization competencies that are quintessential for therapeutic practice. The diagnostic realm too stands to gain, with a potential enhancement in emotion identification methodologies.

Further corroborating the prowess of ChatGPT-4 was its performance on the LEAS, where it manifested an acumen for text-based emotional awareness that superseded human averages. This finding corroborates and is congruent with prior empirical findings [19,21]. Taken in concert, these findings elucidate the multifaceted mentalizing capabilities of ChatGPT-4, span visual and textual modalities, and reinforce previous findings about the potential of LLMs in performing tasks in the mental health field [19,21,30-37]. Additionally, although its nascent visual emotion recognition abilities are noteworthy, its competencies in textual mentalization remain unparalleled, a testament to its foundational architecture rooted in NLP.

However, as the field ventures into this novel territory, prudence is imperative. It must be emphasized that although ChatGPT-4 can simulate emotional understanding on the basis of vast data patterns, it lacks genuine emotional cognition or sentience. Consequently, applications leveraging ChatGPT-4 must be approached with circumspection, ensuring that they neither perpetuate clinical stigmas nor misconstrue AI’s simulated cognition as genuine emotional comprehension.

### Study Limitations

It is crucial to address the limitations of this study for a comprehensive understanding. First, the examination was conducted on specific models at a particular time. Therefore, future updates and versions might yield different results, reflecting the dynamic nature of these models. Second, while the chosen tests effectively measure emotion recognition, they do not capture the full complexity of mentalization, including understanding intentions or other mental states. Third, the study did not examine faces from diverse cultures, ages, or skin tones; the tested images were in black and white, and the norms were based on British and French populations. Furthermore, due to the “black box” nature of these models, it is challenging to ascertain the reasons behind their conclusions and understand the differences between models or iterations within the same model. The opaque nature of the models and the databases on which they were trained make them difficult to pinpoint the exact causes of their successes or shortcomings. Finally, the interaction with ChatGPT and Bard was conducted solely in English, while the norms data for the LEAS used for comparison were collected from a French-speaking general population. This linguistic discrepancy raises concerns about the accuracy and validity of the comparison, as language differences may influence the scores obtained. Nonetheless, it should be noted that the LEAS scores of the normal English-speaking population are similar to the norms of the French-speaking general population [38]. We used the largest available sample of a general population (n=750), which happened to be in French.

### Implications for Responsible AI Development

The study limitations allude to matters of fairness and inclusiveness of the training data as well as to AI model transparency. This underscores the criticality of incorporating a wide-ranging data set in model construction to ensure the representation of a variety of clinical populations and cultural backgrounds. Additionally, the issue of transparency in these models, often termed the “black box” problem due to the unclear nature of their underlying algorithms, poses a significant challenge. Equally critical is the concern regarding the exposure of user data to corporations and the urgent need to adequately address both accessibility and infrastructure for end users [39]. Building on these concerns, attention turns to AI systems with the capacity for human-like emotional recognition. These systems harbor both promise and risk, with opportunities for constructive use in education, patient self-insight, or integration in conversational therapy and diagnosis [19,21]. However, a concern arises that the epistemic authority and credibility afforded to AI via its affective analysis may enable misuse, whether commercial or other, thus acting against patient interests [40]. We recommend mandating disclaimers whenever emotional data are algorithmically processed, enhancing transparency, respecting users’ autonomy, and possibly also mitigating manipulation of users with detected vulnerable states. In addition, given the fundamental human needs for trust and connection, especially in mental health care, it logically follows that improperly developed AI with emotion identification capabilities risks causing harm to people. Safeguarding against this necessitates both mental health experts and patients providing a lived experience perspective in a collaborative development process of these technologies. Given the scale of these systems and their potential outreach, governmental or professional oversight is crucial to safeguard public interests in mental health–related AI advancement. Overall, while showcasing the unique benefits of emotionally intelligent AI, governance is vital to mitigate its risks.

### Conclusions

In conclusion, this research serves as a seminal exploration into the cross-modal mentalization capabilities of AI, especially across visual and textual dimensions. Although the results support for the potential integration of ChatGPT-4 into mental health paradigms, they also underscore the concomitant ethical quandaries that necessitate judicious navigation.

## Abbreviations

AI: artificial intelligence
LEAS: Levels of Emotional Awareness Scale
LLM: large language model
NLP: natural language processing
RMET: Reading the Mind in the Eyes Test

## Data Availability

The data sets generated and analyzed during this study are available from the corresponding author on reasonable request.
